# Herbal Formula SS-1 Increases Tear Secretion for Sjögren’s Syndrome

**DOI:** 10.3389/fphar.2021.645437

**Published:** 2021-09-24

**Authors:** Ching-Mao Chang, Po-Chang Wu, Jr-Rung Lin, Yeong-Jian Jan Wu, Shue-Fen Luo, Yin-Tzu Hsue, Joung-Liang Lan, Tai-Long Pan, Yu-Ting Wu, Kuang-Hui Yu, Yau-Huei Wei, Hen-Hong Chang

**Affiliations:** ^1^ Center for Traditional Medicine, Taipei Veterans General Hospital, Taipei, Taiwan; ^2^ Faculty of Medicine, National Yang Ming Chiao Tung University, Taipei, Taiwan; ^3^ Institute of Traditional Medicine, National Yang Ming Chiao Tung University, Taipei, Taiwan; ^4^ Rheumatology and Immunology Center, China Medical University Hospital, Taichung, Taiwan; ^5^ College of Medicine, China Medical University, Taichung, Taiwan; ^6^ Clinical Informatics and Medical Statistics Research Center, Graduate Institute of Clinical Medical, Chang Gung University, Taoyuan, Taiwan; ^7^ Department of Medicine, Division of Allergy, Immunology, and Rheumatology, Chang Gung Memorial Hospital, College of Medicine, Chang Gung University, Taoyuan, Taiwan; ^8^ Department of Internal Medicine, Division of Rheumatology, Allergy and Immunology, Changhua Christian Hospital, Changhua, Taiwan; ^9^ School of Traditional Chinese Medicine and Graduate Institute of Clinical Medical Sciences, Chang Gung University, Taoyuan, Taiwan; ^10^ Research Center for Chinese Herbal Medicine and Research Center for Food and Cosmetic Safety, College of Human Ecology, Chang Gung University of Science and Technology, Taoyuan, Taiwan; ^11^ Liver Research Center, Chang Gung Memorial Hospital, Taoyuan, Taiwan; ^12^ Center for Mitochondrial Medicine and Free Radical Research, Changhua Christian Hospital, Changhua, Taiwan; ^13^ Department of Biochemistry and Molecular Biology, School of Life Sciences, National Yang Ming Chiao Tung University, Taipei, Taiwan; ^14^ Department of Medicine, Mackay Medical College, Taipei, Taiwan; ^15^ Graduate Institute of Integrated Medicine, College of Chinese Medicine, and Chinese Medicine Research Center, China Medical University, Taichung, Taiwan; ^16^ Department of Chinese Medicine, China Medical University Hospital, Taichung, Taiwan

**Keywords:** Sjögren’s syndrome, Xerophthalmia, Herbal formula, Integrative therapy, SS-1

## Abstract

**Background:** Sjögren’s syndrome (SS) is an autoimmune inflammatory disease that primarily affects the exocrine glands, leading to glandular dysfunction. The hallmark symptoms of SS are dry eyes and mouth, compromising the quality of life of patients and decreasing their capacity to perform their daily activities.

**Objective:** This study aims to evaluate the efficacy of the herbal formula SS-1 for its potential therapeutic benefits for patients with Sjögren’s syndrome.

**Materials and Methods:** The bioactivity profile of SS-1 was determined using four different SS-1 concentrations across 12 human primary cell systems of the BioMAP profile. After that, a randomized, double-blind, crossover, placebo-controlled trial was performed including 57 patients treated with SS-1 for 28 weeks.

**Results:** Biologically multiplexed activity profiling in cell-based models indicated that SS-1 exerted anti-proliferative activity in B cells and promoted anti-inflammatory and immunomodulatory activity. In the clinical trial, Schirmer’s test results revealed significant improvements in both eyes, with increases of 3.42 mm (95% CI, 2.44–4.41 mm) and 3.45 mm (95% CI, 2.32–4.59 mm), respectively, and a significant reduction in artificial tear use, which was −1.38 times/day, 95% CI, −1.95 to −0.81 times/day. Moreover, the increases in B-cell activating factor (BAFF) and B-cell maturation antigen (BCMA) levels were dampened by 53.20% (295.29 versus 555.02 pg/ml) and 58.33% (99.16 versus 169.99 pg/ml), respectively.

**Conclusion:** SS-1 treatment significantly inhibited B-cell maturation antigen. No serious drug-related adverse effects were observed. Oral SS-1 administration may be a complementary treatment for Sjögren’s syndrome.

## Introduction

Sjögren’s syndrome (SS) is an autoimmune inflammatory disease that primarily affects the exocrine glands, leading to glandular dysfunction (Brito-Zeron et al., 2016). The approximate incidence of SS is six to seven per 100,000 individuals (Weng et al., 2011; Qin et al., 2015). Genetic and environmental factors, viral infections, and immune dysregulation are vital in SS’s pathogenesis, because they can cause oxidative stress and inflammation in the lacrimal and salivary glands (Nocturne and Mariette, 2013). Some SS patients have systemic issues such as extra-glandular involvement of pulmonary fibrosis, interstitial nephritis, autoimmune thyroiditis, and lymphoma (Seror et al., 2010; Chatzis et al., 2021).

The interaction of B-cell activating factor (BAFF) and the BAFF receptor as B-cell maturation antigen (BCMA) are important to the pathogenesis of focal lymphocytic infiltration (Daridon et al., 2007; Imgenberg-Kreuz et al., 2018), which can lead to exogland dysfunction of the lacrimal and salivary glands. Thus, the hallmark symptoms of SS are dry eyes and mouth, decreasing patient’s capacity to perform their daily activities and thus compromising their quality of life (Mariette and Criswell, 2018).

The current therapeutic options for SS include hydroxychloroquine for immune system modulation (Saraux et al., 2016). In a recent double-blind, parallel-group, placebo-controlled trial (Gottenberg et al., 2014), 120 patients with primary SS who were treated with hydroxychloroquine showed no improvement after 24 weeks, as measured by the European League Against Rheumatism Sjögren’s Syndrome Patient Reported Index (ESSPRI), Schirmer’s test, and salivary-flow data. Other approaches to treating SS have also failed to achieve significant improvements in tear secretion, measured either by Schirmer’s test (Shih et al., 2017) or by reductions in artificial tear use.

In traditional Chinese medicine (TCM), SS is classified as “dry-bi,” “dryness syndrome,” and “dryness impediment,” and its main pathogenesis factors are theorized to be “yin-deficiency,” “dryness-heat” and “consumption and deficiency of fluid” (Shui-Yan, 2011). Hence, the TCM treatment approach is to “enrich yin, clear heat and moisten dryness,” commonly using Chinese formulas and single herbs that include “Qi-Ju-Di-Huang-Wan,” “Gan-Lu-Yin,” “Xuan-Shen” (*Scrophularia ningpoensis* Hemsl.), and “Mai-Men-Dong” (*Ophiopogon japonicus* (L. f.) Ker-Gawl.). The further the disease progresses, the more fibrotic changes will be observed, i.e., what TCM regards as “stasis” in the exocrine glands. This theoretically can be resolved by “Xue-Fu-Zhu-Yu-decoction” and “Dan-Shan” (*Salvia miltiorrhiza* Bge.), which “quicken the blood and dispel stasis.”

Due to the unsatisfactory effects of conventional medicine, many SS patients have sought herbal-medicine alternatives (Luo et al., 2012; Liu et al., 2016; Feng et al., 2019). However, the efficacy of such treatments as measured by Schirmer’s test have been limited, and the heterogeneity of the studies that have examined them means that no consensus on their optimal application in SS cases has been reached (Luo et al., 2012).

In a previous paper that utilized network analysis (Chang et al., 2015), we identified “Qi-Ju-Di-Huang-Wan,” “Gan-Lu-Yin,” “Xuan-Shen” (*Scrophularia ningpoensis* Hemsl.), “Mai-Men-Dong” (*Ophiopogon japonicus* (L. f.) Ker-Gawl.), and “Sheng-Di-Huang” (*Rehmannia glutinosa* Libosch) as the core components of TCM therapeutic prescriptions for SS in Taiwan. The relevant functions of these components include increased antioxidant capacity, anti-inflammatory effects, and improvement of sicca. However, our analyses indicated that these core-pattern prescriptions did not target the B/T cell activation and fibrosis pathway, which is important in SS pathogenesis. Therefore, we developed the herbal formula SS-1 comprising “Gan-Lu-Yin,” “Sang-Ju-Yin,” and “Xue-Fu-Zhu-Yu decoction” in powder form to test our hypothesis that this combination of components would exhibit antioxidant, anti-inflammatory, immunomodulatory, and anti-fibrotic effects, while also alleviating sicca symptoms (Lin, 2000; Shao, 2008; Fang et al., 2013). We further speculated that 1) “Gan-Lu-Yin” would alleviate mucositis and dry mouth (Lin, 2000), 2) “Xue-Fu-Zhu-Yu decoction” would attenuate fibrotic changes in the salivary glands (Shao, 2008), and 3) “Sang-Ju-Yin” would modulate immune function in patients with SS (Fang et al., 2013).

In another study (Lee et al., 2020), we found that SS-1 functioned to reduce the percentages of CD69^+^ and CD25^+^ cells among CD8^+^ and CD4^+^ T cells upon 24-h activation, as well as the percentages of IL-4^+^ cells and the levels of IL-4 and IL-13 produced by CD4^+^ T cells after T_H_2 polarization. These findings indicated that SS-1 could inhibit both the activation and the T_H_1, T_H_2, and IL-17A^+^IFNγ^+^ T_H_ polarization of murine T cells. Lastly, we found that SS-1 also inhibited the expression of IFN-γ and T_H_2 cytokines, and moderately down-regulated IL-17A expression in SS patients’ peripheral blood mononuclear cells (PBMCs).

To test our hypotheses regarding SS-1’s bioactivity, we utilized *in vitro* biologically multiplexed activity profiling (BioMAP; Eurofns DiscoverX, San Francisco, CA) within a phenotypic profiling platform, with multiplex human primary cell-based assays and a broad panel of translational biomarkers. BioMAP has been extensively employed to profile both compound mechanisms and the secondary activities engaged in by compounds in complex primary human-cell systems (Berg et al., 2006; Williams et al., 2010; Xu et al., 2012; Kleinstreuer et al., 2014).

The first aim of the present study was to determine the bioactivity profiles of four different SS-1 concentrations in more complex primary human-cell systems across 12 BioMAP systems (Berg et al., 2010). Based on our initial findings that all these concentrations exerted anti-proliferative activity in endothelial cells, B cells, and fibroblasts and exhibited good inflammation-related and immunomodulatory activity, we proceeded to conduct a randomized, double-blind, crossover, placebo-controlled clinical trial at two centers to evaluate the safety and efficacy of SS-1 in treating patients with SS.

## Materials and Methods

### BioMAP

The compounds were tested at 0.5×, 0.1×, 0.02×, and 0.004× therapeutic concentrations across 12 human primary-cell systems from the BioMAP Diversity PLUS panel. These were: 3C (venular endothelial cells (VECs) plus Th1), 4H (VECs plus Th2), LPS PBMCs plus VECs for monocyte activation), SAg (PBMCs plus VECs for T-cell activation), BT (PBMCs plus B cells for B-cell activation), BF4T (bronchial epithelial cells (BECs) plus dermal fibroblasts (DFs)), BE3C (BECs), CASM3C (coronary-artery smooth-muscle cells), HDF3CGF (human DFs), KF3CT (DFs plus keratinocytes), MyoF (lung fibroblasts), and Mphg (VECs plus macrophages for macrophage activation) (see Supplementary Table S1). Cell-based enzyme-linked immunosorbent assays were performed to measure the readouts.

### Clinical-Trial Design

This randomized, double-blind, placebo-controlled, crossover, two-center trial was conducted at Chang Gung Memorial Hospital (CGMH) in Taoyuan and China Medical University Hospital (CMUH) in Taichung, both in Taiwan, between February 11, 2014 and April 19, 2016. The first patient with SS was enrolled on July 9, 2014.

This clinical trial was registered at ClinicalTrials.gov (NCT02110446). The relevant Consolidated Standards of Reporting Trials statement and its annex for Chinese herbal medicine (CHM) formulas are provided in the Supplementary Material.

### Ethics Statement

Our clinical trial protocol was reviewed and approved by the institutional review boards of CGMH (102-2481A) and CMUH (CMUH103-REC2-128). Each patient signed an informed consent form before enrolling in the trial.

### Trial Participants

**FIGURE 1:**
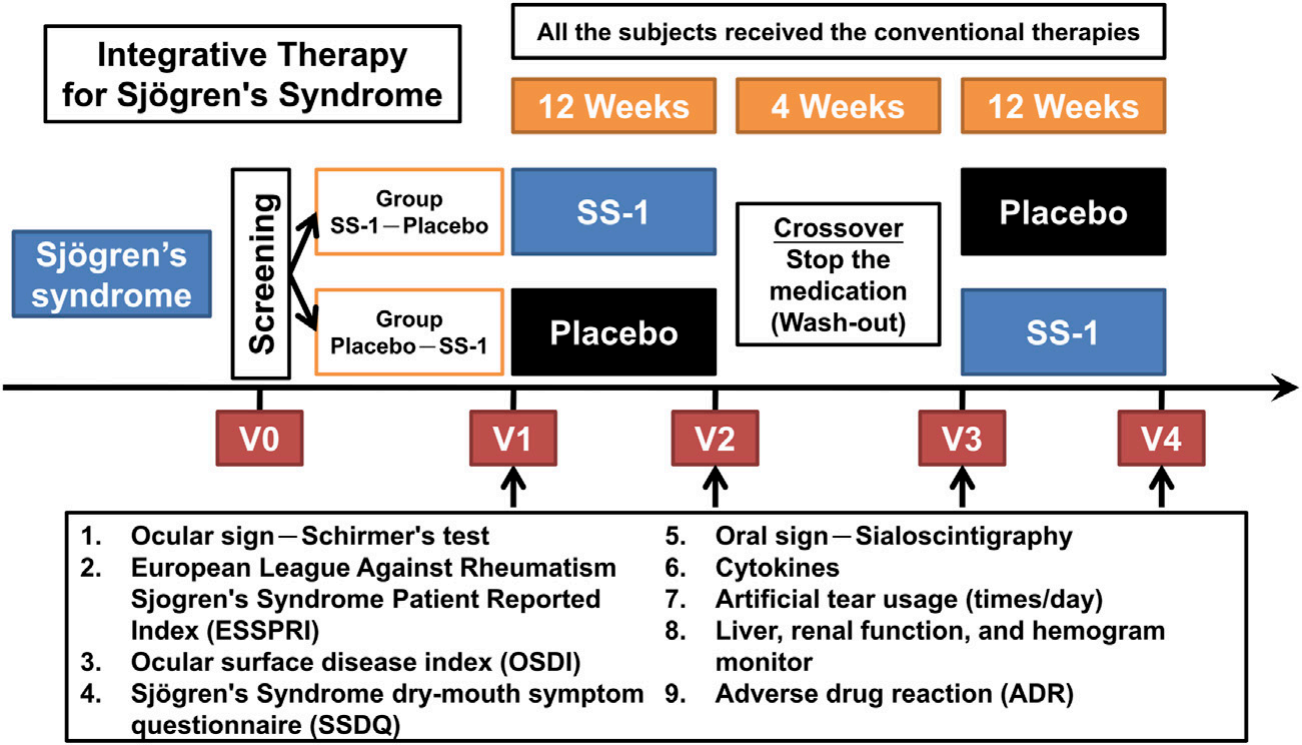
The protocol for this integrated clinical trial. The recruited patients were randomly divided into two groups of “Group SS-1-Placebo” and “Group Placebo-SS-1.” SS-1 was administered in an add-on design, and all the subject’s still required the same HCQ treatment throughout the trial. Both groups did not receive additional pilocarpine or cevimeline, which were administered in the patient’s respective rheumatology OPDs. The “Group SS-1-Placebo” patients first received SS-1 for 12 weeks, after which they underwent a 4-weeks washout period, followed by placebo treatment for 12 weeks; and vice versa for the “Group Placebo-SS-1” patients.

Patients with SS who fulfilled the American-European Consensus Group (AECG) criteria for screening, as revised in 2002, were treated in the rheumatology outpatient departments at CGMH and CMUH. The recruited patients were randomly divided into two groups: SS-1–Placebo and Placebo–SS-1 (Figure 1). All patients continued fixed treatment with hydroxychloroquine throughout the trial, and SS-1 was administered as an add-on therapy. Neither group received additional treatment with either pilocarpine or cevimeline, which were otherwise routinely administered to SS patients by both centers. The patients in the SS-1–Placebo group first received SS-1 for 12 weeks before a 4-weeks washout period, followed by the placebo treatment for 12 weeks. Conversely, the patients in the Placebo–SS-1 group first received placebo for 12 weeks before a 4-weeks washout period, followed by SS-1 treatment for 12 weeks.

### Sjögren’s Syndrome-1 and Placebo Formulations

Each patient in both groups was given 6 g of the test drug three times daily. SS-1 comprised a 2:1:1 ratio of “Gan-Lu-Yin,” “Sang-Ju-Yin,” and “Xue-Fu-Zhu-Yu decoction” in powder form; details of the composition of SS-1 and its certification, previously reported by Chang et al. (2015), Lee et al. (2020) are included in the Supplementary Material. As well as corn starch and pigment, the placebo contained 1/100 of the SS-1 dose used for treatment, which was necessary to mimic the color, taste, and smell of SS-1 due to the double-blind trial design, and as required by the trial’s ethics guidelines. The SS-1 and placebo preparations used in the trial were both manufactured by a reputable pharmaceutical company specializing in the production of compounds for CHM (Kaiser Pharmaceutical Co., Ltd., Taiwan), and the quality of the SS-1 preparation was confirmed using fingerprinting techniques and high-performance liquid chromatography. All data, methods, locations, and collection of experimental SS-1 drug samples were authenticated by licenses.

### Sample-Size Calculation

The desired sample size of 70 subjects was calculated based on an anticipated 35% improvement from the baseline, as measured by Schirmer’s test, with a two-tailed significance level of 0.05% and a power of 0.8. In the event, due to a 25% dropout rate, 52 subjects completed the trial.

### Randomization and Masking

A permuted-block randomization scheme was employed by our team’s statistician (JRL) to generate randomization codes, and equal numbers of patients were assigned to each group. The randomization table was then sent to the pharmaceutical company that manufactured the SS-1 and placebo formulations, which were then sent to the departments of Traditional Chinese Medicine Pharmacy at CGMH and CMUH. Due to the double-blind trial design, none of the patients, doctors, pharmacists, care providers, or investigators knew which patients were in which group. After the trial was completed, the randomization was decoded to facilitate statistical analysis.

### Inclusion Criteria

All patients enrolled in the trial fulfilled the following inclusion criteria: 1) having a diagnosis of primary or secondary SS, with the latter managed for at least 3 months prior to enrollment via a consistent treatment comprising prednisolone (≤10 mg/day) and a fixed hydroxychloroquine dose; 2) being between 20 and 75 years old; 3) meeting the AECG criteria for SS, as revised in 2002; 4) in the case of patients receiving cyclosporine, cevimeline, pilocarpine, rituximab, or other biological agents, consenting to discontinue treatment 1 month before trial enrollment and to fully cooperate until the trial’s end; 5) consenting to the cessation of any non-trial-related treatment with “Gan-Lu-Yin,” “Sang-Ju-Yin,” or “Xue-Fu-Zhu-Yu decoction” 1 month before entering the trial; and 6) lacking any abnormal hemogram findings regarding liver and kidney function.

### Exclusion Criteria

The exclusion criteria were: 1) any history of alcohol abuse, diabetes mellitus, or major life-threatening conditions; 2) currently pregnant or breastfeeding; 3) currently having abnormal liver or kidney functions; and 4) having had steroid pulse therapy within 3 months prior to the commencement of the trial.

### Outcome Measures

The primary endpoints in this trial were the results of the Schirmer’s test, ESSPRI, SS Dry Mouth Symptom Questionnaire (SSDQ), Ocular Surface Disease Index (OSDI), and sialoscintigraphy (Ramos-Casals et al., 2020). The Schirmer’s test is an objective evaluation of tear secretion, in which a result ≦5 mm represents dry eye (Vitali et al., 2002). ESSPRI is a self-report instrument in which patients rate their dryness, fatigue, and pain on a scale from 0 to 10, with score of 0 indicating that the issue is not present, and 10 that it is maximal (Seror et al., 2011). SSDQ is another self-report instrument, commonly administered in Taiwan to patients complaining of dry mouth. Its scores range from −10 to 30, with a score of 10 or higher indicating that a drug taken to alleviate dry mouth has been efficacious (Wu et al., 2006). A third self-report instrument, the OSDI, contains 12 subdomains covering ocular symptoms, daily activities, and environmental factors. The score for each subdomain ranges from 0 to 4, with 0 representing no symptoms and 4, continuous symptoms. A person’s total OSDI score can therefore range from 0 to 100, with higher scores indicating more severe disease and more profound effects on vision-related functions (Schiffman et al., 2000). Sialoscintigraphy is an objective evaluation of salivary-gland functions, and produces a score ranging from 0 to 4. In the current trial, bilateral parotid glands were evaluated using this technique, and scores of 0, 1, 2, 3, and 4 indicated prominent, moderate, mild, minimal, and no uptake or excretion, respectively (Wu et al., 2006).

The trial’s secondary endpoints were 1) self-reported numbers of artificial-tear applications per day, ranging from 0 to 30 or more, with more use indicating more eye dryness; 2) oxidative stress and antioxidant capacity; 3) quality of life (SF-36); and 4) regulatory effects on cytokines. We also monitored drug safety by evaluating drug use, adverse drug effects, adverse events (AEs), serious AEs (SAEs), biochemical test results, and hemogram results.

We evaluated the above parameters at four time-points. These were: immediately before treatment (V1), after 12 weeks of treatment (V2), at the end of the washout period (V3), and at the completion of crossover treatment (V4) (Figure 1).

### Statistical Analysis

We computed the participant’s baseline characteristics as means ± standard deviation. Their demographic data and medical conditions were analyzed via chi-squared tests, in the case of univariate comparisons between categorical variables, and via *t*-tests, for continuous variables. The trial outcomes were analyzed using the per-protocol model, and outcome differences between the SS-1–Placebo and Placebo–SS-1 groups were evaluated through analysis of variance in a 2 × 2 crossover design. Treatment outcomes for each group were presented as means with 95% confidence intervals (CIs) for crossover analysis. All statistical analyses were performed using SAS software version 9.3 (SAS Institute, Cary, NC). All tests were two-tailed, and *p* values < 0.05 were considered to indicate statistical significance.

## Results

### BioMAP Results

**FIGURE 2:**
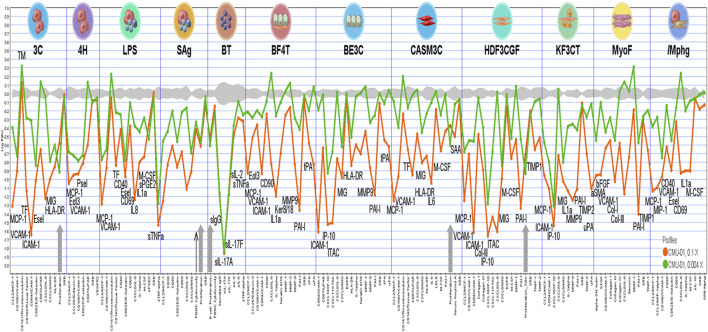
The analysis of bioactivities in each BioMAP assay systems. The cytotoxic and anti-proliferative activities are illustrated by thin black arrow and grey arrows, respectively.

The bioactivity analysis in each BioMAP assay system, as shown in Figure 2, indicated that SS-1 was associated with anti-proliferative activity in endothelial cells, B cells, and fibroblasts (gray arrows). SS-1 also exhibited inflammation-related, immunomodulatory, and tissue remodeling activity. Specifically, it led to significant reductions in the following 16 biomarkers for inflammation-related activity: monocyte chemotactic protein 1, vascular cell adhesion protein 1, intercellular adhesion molecule 1, E-selectin, monokine induced by gamma interferon, eotaxin-3, P-selectin, IL-8, IL-1α, secreted prostaglandin E2, secreted tumor necrosis factor-α, interferon gamma-inducible protein 10, interferon-inducible T-cell α chemoattractant, IL-6, serum amyloid A, and macrophage inflammatory protein 1-α. Similarly, SS-1 significantly reduced the levels of the following eight biomarkers for immunomodulatory-related activity: human leukocyte antigen-DR, cluster of differentiation (CD) 40, CD69, macrophage colony-stimulating factor, secreted immunoglobulin G, secreted IL-17A (sIL-17A), secreted IL-17F, and secreted IL-2. Lastly, SS-1 was able to significantly reduce the levels of the following 12 biomarkers for tissue remodeling-related activity: CD90, keratin 8/18, matrix metalloproteinase 9, plasminogen activator inhibitor 1, tissue plasminogen activator, collagen I, collagen III, tissue inhibitor of metalloproteinase 1, tissue inhibitor of metalloproteinase 2, urokinase plasminogen activator, α-smooth muscle actin, and basic fibroblast growth factor. Specifically, sIL-17A–a proinflammatory cytokine that induces cytokine production and mediates monocyte and neutrophil recruitment to sites of inflammation indicates immunomodulation-related activity in the BT system, modulating T-cell-dependent B-cell activation. As shown in Figure 2, sIL-17A was associated with the strongest reduction in the BT system.

### Clinical Trial Population and Interventions

**FIGURE 3:**
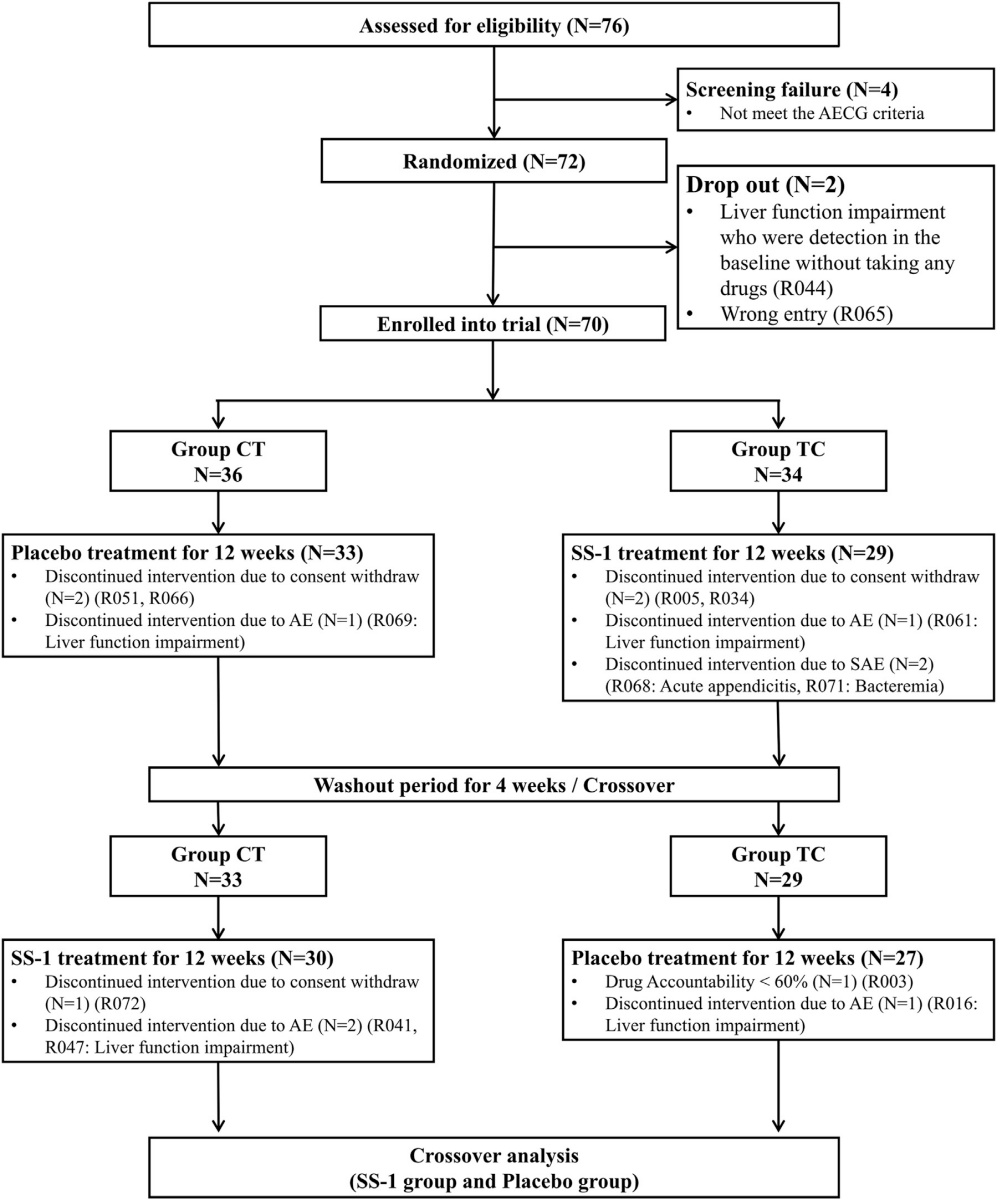
Flowchart of recruitment of patients with Sjögren’s syndrome in this integrated clinical trial.

Of 76 patients with SS who were screened, four did not meet the 2002 AECG criteria and were excluded. Two further patients subsequently dropped out because of impaired liver function, detected at baseline in the absence of drug treatment, and who had therefore been enrolled erroneously (i.e., had not in fact met the 2002 AECG criteria). Thus, a total of 70 patients remained for randomization, of whom 36 were placed in the Placebo–SS-1 group, and the other 34 in the SS-1–Placebo group. The former group included two patients with systemic lupus erythematosus, and the latter, two with that condition and one with systemic sclerosis. During the trial period, six members of the Placebo–SS-1 group and seven of the SS-1–Placebo group left the trial by their own request or due to AEs, SAEs, and low drug accountability. After 28 weeks of treatment, the crossover analysis proceeded with the 30 and 27 patients who remained in the Placebo–SS-1 and SS-1–Placebo groups, respectively (Figure 3).

The baseline characteristics of the patients are listed in Table 1. Briefly, 94 and 97%, respectively, of those in the initial Placebo–SS-1 and SS-1–Placebo groups were female. The mean time interval between an individual’s SS diagnosis and enrollment was 3.9 ± 4.0 years in the Placebo–SS-1 group, and 4.8 ± 4.2 years in the SS-1–Placebo group. The two groups’ means for artificial tear use were 2.8 ± 1.8 times/day and 3.0 ± 2.3 times/day, and their rates of positivity for rheumatoid factor were 25.0 and 32.3%, respectively. Conversely, their rates of positivity for anti-Ro were 77.8 and 73.5%, and for anti-La, 38.9 and 29.4%, respectively. None of the baseline characteristics differed significantly between the two groups, and none of the patients received cyclosporine, cevimeline, pilocarpine, rituximab, or other biological agents before enrolling in this trial.

**TABLE 1:** Baseline characteristics of the SS-1 trial for Sjögren’s syndrome patients.

	Group Placebo-SS-1 (N = 36)	Group SS-1-Placebo (N = 34)	p
Female sex — no. of patients (%)	34 (94)	33 (97)	
Age, year	52.5 ± 12.3	51.7 ± 10.9	0.775
Time to diagnosis, year	3.9 ± 4.0	4.8 ± 4.2	0.376
Conventional medicine			
Hydroxychloroquine	245.8 ± 83.3	245.8 ± 83.3	1
Artificial tear usage (times/day)	2.8 ± 1.8	3.0 ± 2.3	0.718
Outcome			
Schirmer’s test (mm)^a^			
OS (left eye)	3.3 ± 4.6	3.1 ± 3.5	0.824
OD (right eye)	2.7 ± 3.5	3.3 ± 5.7	0.580
ESSPRI Score^b^			
Dryness	6.3 ± 1.8	5.8 ± 2.6	0.321
Fatigue	6.1 ± 1.8	5.6 ± 2.6	0.345
Pain	5.0 ± 3.0	4.2 ± 3.1	0.276
OSDI Score^c^	49.8 ± 21.7	46.8 ± 24.4	0.560
Eyes that are sensitive to light	2.4 ± 1.3	2.4 ± 1.4	0.775
Eyes that feel gritty	2.1 ± 1.4	1.9 ± 1.2	0.580
Painful or sore eyes	1.4 ± 1.3	1.3 ± 1.1	0.812
Blurred vision	2.3 ± 1.3	1.2 ± 1.3	0.144
Poor vision	2.1 ± 1.3	1.9 ± 1.4	0.586
Reading	2.0 ± 1.3	1.9 ± 1.4	0.856
Driving at night	1.1 ± 1.3	1.1 ± 1.6	0.921
Working with a computer or bank machine (ATM)	1.5 ± 1.5	1.5 ± 1.5	0.996
Watching TV	1.6 ± 1.3	1.8 ± 1.3	0.608
Windy conditions	2.4 ± 1.3	2.1 ± 1.5	0.326
Places or areas with low humidity (very dry)	2.2 ± 1.2	1.9 ± 1.3	0.304
Areas that are air conditioned	2.0 ± 1.2	1.9 ± 1.4	0.851
Sialoscintigraphy			
Uptake	3.0 ± 1.0	3.0 ± 1.1	1.000
Excretion	1.8 ± 1.1	1.4 ± 1.4	0.140
Laboratory data			
White blood cells (103/µL)	5.2 ± 1.2	4.9 ± 1.9	0.511
Red blood cells (106/µL)	4.4 ± 0.47	4.5 ± 0.6	0.209
Hemoglobin (g/dl)	13.1 ± 1.1	12.9 ± 1.1	0.470
Platelet (103/µL)	219.7 ± 51.9	221.7 ± 49.1	0.872
Aspartate aminotransferase (µ/L)	25.6 ± 7.4	24.6 ± 4.8	0.531
Alanine aminotransferase (µ/L)	20.1 ± 4.4	18.9 ± 9.7	0.591
Blood urea nitrogen (mg/dl)	11.9 ± 3.4	12.7 ± 4.1	0.381
Serum creatinine (mg/dl)	0.7 ± 0.2	0.6 ± 0.1	0.078
Erythrocyte sedimentation rate (mm/hr)	17.7 ± 18.4	15.9 ± 10.8	0.625
Positive rates — no. of patients (%)			
Rheumatoid factor (RF)	9 (25.0)	11 (32.3)	
Anti-Ro	28 (77.8)	25 (73.5)	
Anti-La	14 (38.9)	10 (29.4)	
Anti-Mitochondrial	0 (0)	2 (5.8)	
Cytokines (pg/ml)			
IL-1β	742.27 ± 1,141.66	572.95 ± 550.63	0.754
IL-17A	42.99 ± 112.73	290.34 ± 1,010.63	0.188
IL-18	149.01 ± 109.62	196.51 ± 255.12	0.323
IL-23	100.97 ± 207.90	23.17 ± 25.07	0.344
IL-27	721.87 ± 1,182.47	849.23 ± 1,683.65	0.809
MMP-9	34,514.58 ± 23,578.48	43,423.26 ± 41,234.96	0.278
IFN-α	84.07 ± 44.87	16.36 ± 0.00	0.321
IFN-γ	42.74 ± 28.23	43.09 ± 37.37	0.967
BAFF	1,331.00 ± 839.79	1,480.14 ± 1,363.08	0.581
BCMA	293.07 ± 195.20	301.22 ± 173.64	0.854

SS, Sjögren's syndrome; ESSPRI, EULAR Sjogren's Syndrome Patient Reported Index; OSDI, ocular surface disease index; MMP-9, matrix metalloproteinase-9; BAFF, B-cell-activating factor; BCMA, B-cell-maturation antigen; IL, interleukin; IFN, interferon. The baseline demographic data and medical conditions were analyzed using ANOVA test for continuous variables, and the baseline characteristics data in the text and tables are expressed as mean ± standard deviation (SD). And there were no significant differences in the baseline characteristics between the two treatment groups. ‖Artificial tear usage: a record which the patient self-reported ranged from 0 to 30 or much more times/per day, the more usage represents more eye dryness.

a Schirmer’s test: an objective evaluation of tear secretion, ranged from 0 to 30 mm, in which less than 5 mm represents dry eye, and more the value represents much more tear secretion).

b ESSPRI Score: European League Against Rheumatism Sjögren’s Syndrome Patient Reported Index is a patient self-reported questionnaire about dryness, fatigue, and pain, which it ranged from 0 to 10 for each item; while 0 represents no dryness, fatigue or pain; and 10 represents the maximal dryness, fatigue or pain.

c OSDI Score: Ocular surface disease index is a patient self-reported questionnaire containing 12 sub-domains about ocular symptoms, daily activities, and environment factors, which each sub-domain ranged from 0 to 4; while 0 represents none, and 4 represents all of the time. And the total score of OSDI ranged from 0 to 100, while the higher scores represent greater dry eye disease severity and effect on vision-related functions.

Sialoscintigraphy: an objective evaluation of salivary gland functions, ranged from 0 to 4, bilateral parotid glands were evaluated as 0 represents prominent uptake or excretion; 1 represents moderate uptake or excretion; 2 represents mild uptake or excretion; 3 represents little uptake or excretion; 4 represents no uptake or excretion.

### Schirmer’s Test

**FIGURE 4:**
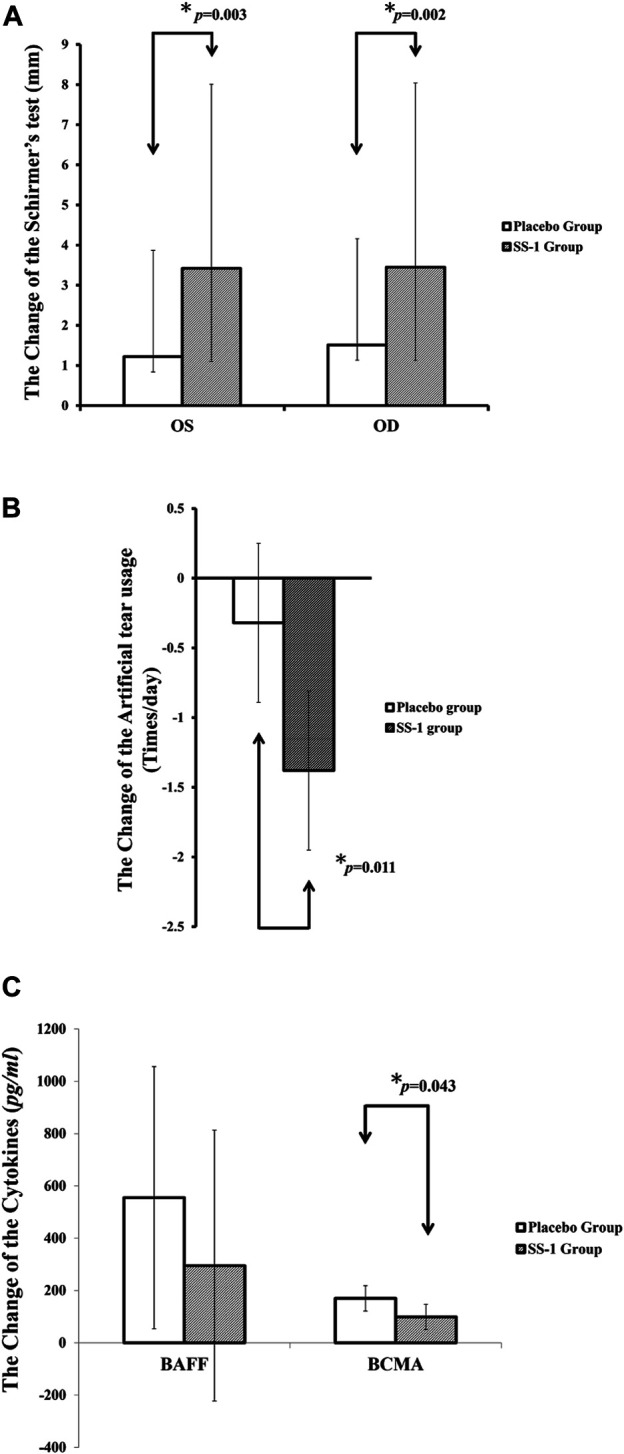
The outcome improvements in **(A)** Schirmer’s test, **(B)** Artificial tear usage after the integrated treatment, and **(C)** serum levels of BAFF and BCMA after the integrated treatment.

Crossover analysis (Table 2) of the Schirmer’s test results revealed that, after SS-1 treatment, the values for the participant’s left and right eyes increased by 3.42 mm (95% CI, 2.44–4.41 mm, *p* = 0.003) and 3.45 mm (95% CI, 2.32–4.59 mm, *p* = 0.002), respectively. Following 12 weeks of placebo treatment, the parallel observed increases were 1.17 mm (95% CI, 0.20–2.14 mm) and 1.51 mm (95%CI, 0.38–2.65 mm) (Figure 4A). Schirmer’s test results were also analyzed via a 2 × 2 crossover design with the original values. This revealed that, after SS-1 treatment, the left- and right-eye values were respectively 5.74 mm (95% CI, 4.84–6.63 mm, *p* = 0.005) and 6.37 mm (95% CI, 5.35–7.38 mm, *p* = 0.008), as against 3.89 mm (95% CI, 3.00–4.79 mm) and 4.39 mm (95% CI, 3.38–5.41 mm) after placebo treatment.

**TABLE 2:** The clinical outcome of the SS-1 trial for Sjögren’s syndrome patients.

	Placebo group	SS-1 group	*P*
Schirmer’s test (mm)^c^			
OS (left eye)	1.22 (0.23–2.21)	3.42 (2.44–4.41)	0.003^a^
OS (left eye)^b^	3.89 (3.00–4.79)	5.74 (4.84–6.63)	0.005^a^
OD (right eye)	1.51 (0.38–2.65)	3.45 (2.32–4.59)	0.002^a^
OD (right eye)^b^	4.39 (3.38–5.41)	6.37 (5.35–7.38)	0.008^a^
ESSPRI Score^d^			
Dryness	−0.71 (−1.25 to −0.18)	−0.95 (−1.48 to −0.95)	0.537
Fatigue	−0.84 (−1.43 to −0.26)	−0.93 (−1.52 to −0.35)	0.827
Pain	−0.59 (−1.33 to 0.15)	0.11 (−0.63 to 0.85)	0.185
OSDI Score^e^	−5.72 (−10.78 to −0.67)	−7.98 (−13.03 to −2.93)	0.530
Eyes that are sensitive to light	−0.33 (−0.70 to 0.05)	−0.39 (−0.76 to −0.01)	0.822
Eyes that feel gritty	−0.10 (−0.41 to 0.21)	−0.11 (−0.42 to 0.20)	0.953
Painful or sore eyes	−0.23 (−0.54 to 0.07)	−0.14 (−0.44 to 0.17)	0.648
Blurred vision	−0.30 (−0.64 to 0.04)	−0.21 (−0.55 to 0.13)	0.696
Poor vision	−0.26 (−0.68 to 0.16)	−0.28 (−0.70 to 0.14)	0.945
Reading	−0.17 (−0.54 to 0.20)	−0.42 (−0.79 to −0.05)	0.336
Driving at night	0.05 (−0.27 to 0.36)	−0.19 (−0.51 to 0.12)	0.287
Working with a computer or bank machine (ATM)	−0.23 (−0.57 to 0.11)	−0.24 (−0.58 to 0.10)	0.981
Watching TV	−0.16 (−0.45 to 0.13)	−0.55 (−0.84 to −0.26)	0.062
Windy conditions	−0.30 (−0.62 to 0.03)	−0.31 (−0.63 to 0.02)	0.963
Places or areas with low humidity (very dry)	−0.31 (−0.65 to 0.03)	−0.55 (−0.89 to −0.21)	0.324
Areas that are air conditioned	−0.30 (−0.64 to 0.05)	−0.52 (−0.86 to −0.17)	0.371
SSDQ Score^f^	9.36 (8.49–10.22)	10.29 (9.43–11.15)	0.173
Sialoscintigraphy			
Uptake	−0.32 (−0.84 to 0.20)	−0.60 (−1.12 to 0.08)	0.453
Excretion	−0.11 (−0.24 to 0.01)	0.01 (−0.11 to 0.14)	0.150
Artificial tear usage (times/day)	−0.32 (−0.89 to 0.25)	−1.38 (−1.95 to −0.81)	0.011^a^
Cytokines (pg/ml)			
IL-17A	17.60 (−106.83 to 142.04)	−6.62 (−126.34 to 113.09)	0.779
IL-18	35.53 (−7.08 to 78.14)	16.75 (−26.83 to 60.32)	0.539
IL-23	219.10 (10.10–428.09)	64.09 (−238.41 to 366.58)	0.349
IL-27	−143.36 (−1,392.53 to 1,105.81)	−501.01 (−1,675.93 to 673.92)	0.675
MMP-9	11,517.00 (−3,990.98 to 27,025.00)	−2,592.48 (−16545.00 to 11,360.00)	0.180
BAFF	555.02 (53.95–1,056.09)	295.29 (−222.46 to 813.03)	0.473
BCMA	169.99 (121.65–218.32)	99.16 (50.83–147.50)	0.043^a^

The data are presented as mean and 95% confidence interval with crossover analysis.

a p<0.05, which these variables were calculated as change from baseline.

b Schirmer’s test of OS (left eye) and OD (right eye) were analyzed by 2×2 crossover design with original value‖Artificial tear usage: a record which the patient self-reported, ranged from 0 to 30 or much more times/per day, the more usage represents more eye dryness.

c Schirmer’s test: an objective evaluation of tear secretion, ranged from 0 to 30 mm, in which less than 5 mm represents dry eye, and more the value represents much more tear secretion).

d ESSPRI Score: European League Against Rheumatism Sjögren’s Syndrome Patient Reported Index is a patient self-reported questionnaire about dryness, fatigue, and pain, which it ranged from 0 to 10 for each item; while 0 represents no dryness, fatigue or pain; and 10 represents the maximal dryness, fatigue or pain.

e OSDI Score: Ocular surface disease index is a patient self-reported questionnaire containing 12 sub-domains about ocular symptoms, daily activities, and environment factors, which each sub-domain ranged from 0 to 4; while 0 represents none, and 4 represents all of the time. And the total score of OSDI ranged from 0 to 100, while the higher scores represent greater dry eye disease severity and effect on vision-related functions.

f SSDQ scores: Sjögren’s Syndrome dry-mouth symptom questionnaire is a patient self-reported questionnaire about dry month using in Taiwan, which it ranged from −10 to 30, in which the scores over 10 represent the efficacy of this drug for improving dry month.

Sialoscintigraphy: an objective evaluation of salivary gland functions, ranged from 0 to 4, bilateral parotid glands were evaluated as 0 represents prominent uptake or excretion; 1 represents moderate uptake or excretion; 2 represents mild uptake or excretion; 3 represents little uptake or excretion; 4 represents no uptake or excretion.

### Artificial Tear Use

As shown in Figure 4B, artificial tear use decreased significantly more in patients treated with SS-1 (−1.38 times/day, 95% CI, −1.95 to −0.81 times/day, *p* = 0.011) than in those treated with the placebo (−0.32 times/day, 95% CI, −0.89 to 0.25 times/day).

### Clinical Parameters

As compared to placebo treatment (OSDI: −5.72, 95% CI:−10.78 to 0.67; SSDQ: 9.36, 95% CI:8.49-10.22; sialoscintigraphy: −0.11, 95% CI:−0.24 to 0.01), SS-1 resulted in a more pronounced decrease in OSDI scores (−7.98, 95% CI: −13.03 to 2.93), a more marked increase in SSDQ scores (10.29, 95% CI: 9.43–11.15), and a greater increase in excretion as measured by sialoscintigraphy (0.01, 95% CI: −0.11 to 0.14).

### Changes in Cytokine Levels

After SS-1 treatment, as shown in Figure 4C, the patients’ BAFF was 295.29 pg/ml (95% CI, −222.46 to 813.03 pg/ml) and their BCMA was 99.16 pg/ml (95% CI, 50.83–147.50 pg/ml, *p* = 0.043). Both these figures were significantly lower than those of patients who received placebo treatment (BAFF, 555.02 pg/ml, 95% CI, 53.95–1,056.09 pg/ml; BCMA, 169.99 pg/ml, 95% CI, 121.65−218.32 pg/ml). In other words, SS-1 treatment dampened increases in BAFF levels by 53.20% (295.29 versus 555.02 pg/ml) and BCMA levels by 58.33% (99.16 versus 169.99 pg/ml), as compared to the placebo.

SS-1 recipients also experienced decreases in their levels of IL-17A (−6.62, 95% CI: −126.34–113.09) and MMP-9 (−2,592.48, 95% CI: −16,545.00–11,360.00), while increases in their IL-18 and IL-23 levels were dampened by 47.14% (SS-1/Placebo: 16.74/35.53) and 29.25% (SS-1/Placebo: 64.09/219.1) as compared with placebo recipients.

### Baseline Characteristics and Outcomes of Patients With Primary Sjögren’s Syndrome

Of the subjects who completed the trial, 65 had primary SS and the other five, secondary SS. Supplementary Tables S2, S3 summarize our analysis of the primary-SS cohort’s baseline characteristics and outcome measures. Schirmer’s testing of these patients revealed that, when treated with SS-1, their left- and right-eye values were respectively increased by 3.63 mm (95% CI, 2.58–4.68 mm, *p* = 0.002) and 5.74 mm (95% CI, 4.84–6.63 mm, *p* = 0.005), i.e., by much more than their placebo-group counterparts (left eye, 1.23 mm, 95% CI, 0.18–2.27 mm; right eye, 3.89 mm, 95% CI, 3.0–4.79 mm). Additionally, treatment with SS-1 led to significant improvements in OSDI sub-scores: for instance, in the “Watch TV” category, where the change was significantly greater (−0.52, 95% CI, −0.79 to −0.25, *p* = 0.048) than for the primary-SS members of the placebo group (−0.14, 95% CI, −0.41 to 0.13). However, the BCMA did not have the significance between two groups.

In addition, the decrease in artificial tear use among patients receiving SS-1 treatment (−1.50 times/day, 95% CI, −2.10 to −0.90 times/day) was significantly greater, *p* = 0.010, than among those receiving the placebo (−0.36 times/day, 95% CI, −0.96 to 0.25 times/day).

### Adverse Events and Serious Adverse Events

During the trial, 12 AEs and two SAEs were reported (Table 3). Five of the AEs involved elevated serum transaminase levels; among these five occurrences, the probable cause was potentially linked to SS-1 in only one, given that the others took place either during placebo treatment or before treatment initiation. The remaining seven AEs were itchy skin, dysuria, upper abdominal pain, herpes simplex, acute conjunctivitis, urinary-tract infection, and common cold. One of the SAEs was acute appendicitis, which was unlikely to be related to SS-1, and the other was bacteremia, which was conclusively unrelated to SS-1.

**TABLE 3:** The adverse event and severe adverse events of the SS-1 trial for patients with Sjögren’s syndrome.

Adverse event	Number	Association
Elevation of serum transaminases	5	1: probable related 2: placebo period 1: definitely not related (Not yet taking the drug)
		1: unlikely related
		
		
Itchy skin	1	unlikely related
Dysuria	1	placebo period
Upper abdominal pain	1	unlikely related
Herpes simplex	1	definitely not related
Acute conjunctivitis	1	placebo period
Urinary tract infections	1	unlikely related
Common cold	1	placebo period
Severe adverse event		
Acute appendicitis	1	unlikely related
Bacteremia	1	definitely not related

## Discussion

### Main Findings and Comparisons With the Literature

Translational research approaches using *in vitro* BioMAP systems were followed by Taiwan’s first clinical trial to evaluate the therapeutic efficacy and potential underlying mechanism(s) of SS-1 for the treatment of SS. Schirmer’s test results and the self-reported incidence of artificial tear use both indicated that SS-1 improved the secretory function of lacrimal glands and significantly alleviated dry-eye symptoms in SS patients.

Current treatments for SS commonly involve the use of artificial tears to relieve eye dryness (Ramos-Casals et al., 2012; Moutsopoulos, 2014). Although hydroxychloroquine is often prescribed for SS, it cannot meaningfully improve either subjective or objective dry-eye symptoms (Akpek et al., 2011; Gottenberg et al., 2014).

Prior claims have been made that some CHMs can improve Schirmer’s test results (Luo et al., 2012; Liu et al., 2016; Feng et al., 2019); however, the reported effects were marginal, and the methodologies of the relevant studies did not meet the CONSORT criteria. In this study, our Schirmer’s test results indicated that our CHM formulation was more efficacious than previously reported ones, while our use of crossover analysis facilitated examination of the differential effects of that formulation and a placebo. Specifically, we found that SS-1 could increase Schirmer’s test results for the left and right eyes to 3.42 mm (95% CI, 2.44–4.41 mm, *p* = 0.003) and 3.45 mm (95% CI, 2.32–4.59 mm, *p* = 0.002), respectively; as compared to just 1.17 mm (95% CI, 0.20–2.14 mm) and 1.51 mm (95% CI, 0.38–2.65 mm) in the placebo group. Moreover, the degree of improvement in OSDI scores was greater for the SS-1 group than for the placebo group, suggesting that oral SS-1 can stimulate tear production.

### Secondary Outcomes and Comparisons With the Literature

The SS-1 group also exhibited sharper increases in SSDQ scores and sialoscintigraphy salivary-excretion results than the placebo group did. In Taiwan, where SSDQ is used to evaluate the therapeutic efficacy of pilocarpine and cevimeline, an SSDQ-score increase of more than 10 is widely held to indicate that a treatment has been effective. Our use of SS-1 increased such scores by an average of 10.29, and can thus be deemed useful in the relief of dry-mouth symptoms. Although oral pilocarpine or cevimeline treatment can also relieve such symptoms, some patients on the latter two medications have experienced side effects including palpitations, sweating, and gastrointestinal disturbances (Noaiseh et al., 2014; Cheng et al., 2016). It is reasonable to expect that if SS-1 were used in combination with lower doses of pilocarpine or cevimeline, those side effects would be reduced, while overall therapeutic efficiency might increase.

Among the pathological mechanisms underlying SS, BAFF and BCMA are considered vitally important (Gross et al., 2000; Nocturne and Mariette, 2013; Vincent et al., 2013; Nezos et al., 2014), particularly in patients with advanced disease, given that BAFF-BCMA interaction may promote lymphocyte proliferation (Jiang et al., 2011; Cornec et al., 2012). Our results suggest that SS-1 may regulate the levels of both BAFF and BCMA, resulting in the down-regulation of lymphocyte activity. However, the result of BCMA in only primary SS had the similar tendency with all SS, while the BCMA did not have the significance between two groups. The activated BAFF and BCMA (Kim et al., 2011; Vincent et al., 2013) may be the main potential pathogenesis of secondary SS, as four subjects with systemic lupus erythematosus and one with systemic sclerosis.

IL-17A (Nguyen et al., 2008; Furuzawa-Carballeda et al., 2014; Li et al., 2016) and MMP-9 (Petznick et al., 2013; Aluri et al., 2015) are highly expressed in the lacrimal and salivary glands. IL-17A may be related to inflammation in dacryoadenitis (Jie et al., 2010; Tan et al., 2014) and in the salivary glands (Fei et al., 2014), while MMP-9 may be involved in the early phase of dry-eye syndrome and tissue fibrosis (Sambursky et al., 2013; Lopez-Miguel et al., 2016). Based on our trial results, SS-1 seems to reduce IL-17A and MMP-9 levels, suggesting that this drug could reduce inflammation in the lacrimal and salivary glands, and be anti-fibrotic.

IL-18 has been linked to the severity of SS (Chen et al., 2016), inflammation (Baldini et al., 2013), parotid-gland enlargement (Tzioufas et al., 2012), and lymphoma (Ciccia et al., 2015), while IL-23 appears to promote differentiation of Th17 cells (Katsifis et al., 2009; Jin and Yu, 2013). Among the patients in our trial who received SS-1, increases in IL-18 levels were less than half (47.14%) and in IL-23 levels, less than a third (29.25%), of the parallel increases observed in the placebo group. Thus, SS-1 may regulate IL-18 and IL-23 levels, decreasing the risk of lymphoma, inflammation, and Th17-cell differentiation. Moreover, SS-1 could down-regulate expression of Th17-pathway-related inflammatory cytokines, including IL-17A, IL-18, and IL-23.

SS patients tend to have higher IL-27 levels than healthy subjects, and such levels in SS patients with interstitial lung disease are higher than those of SS patients without it (Xia et al., 2012). In our study, IL-27 expression was more inhibited by SS-1 than by the placebo, indicating that SS-1 could alleviate inflammation and fibrosis. However, some studies have reported that IL-27 can inhibit inflammation and the activation of Th17 cells (Meka et al., 2015; Zhu et al., 2015). Further studies are thus required to clarify our understanding of SS-1’s therapeutic mechanisms.

### The Mechanism of Sjögren’s Syndrome-1 From the BioMAP

In our BioMAP results, SS-1 exhibited a dose-dependent cytotoxicity in T cells, good anti-proliferative activity in B cells and fibroblasts, and good inflammation-related, immunomodulatory, and tissue-remodeling activities.

Histopathologically, Sjögren syndrome mainly features extensive CD4^+^ T cells and antibody-secreting B cell infiltration, which together cause the destruction of salivary glands (Fox, 1996). Dendritic cells are also found near the ductal epithelium, where they secrete inflammatory cytokines such as TNF-α and IL-6 (O'Dell, 2020). TNF-α induces apoptosis of salivary-gland cells (Jin and Yu, 2013), while IL-6 participates in the generation of Th17 cells (Youinou and Pers, 2011). T cells that secrete IL-17 have been widely detected in diseased salivary glands, suggesting that the pathogenesis of SS may in part be Th17 development (Brito-Zeron et al., 2016). In the presence of IL-6, Th17 cells also orchestrate the development of germinal centers (GCs) (Hsu et al., 2008), which facilitate antigen-driven B cell responses in the salivary glands (O'Dell, 2020).

Our BioMAP results also reveal that SS-1 exhibits a dose-dependent cytotoxicity in T cells, and good anti-proliferative activity in B cells, both of which may benefit salivary glands affected by SS. SS-1 was also shown to have good anti-inflammatory properties, including significant reductions in secreted TNF-α, IL-6, and sIL-17A. Since TNF-α, IL-6, and IL-17 all play important roles in SS pathogenesis, SS-1 can reasonably be expected to have good therapeutic effects on patients affected by SS. Our formulation also displays immunomodulatory properties–including significant down-regulation of HLA-DR, CD40, and CD69 expressions in immune cells–that hamper SS’s dendritic-cell-mediated T cell activation.

### Novelty and Significance of This Study

Current therapeutic options for SS are limited, and the drugs used have had adverse effects on some patients. One recent study used hydroxychloroquine to treat 120 patients with primary SS in a double-blind, parallel-group, placebo-controlled trial, but found no improvement after 24 weeks, as measured by the ESSPRI, Schirmer test results, and salivary-flow data.

Our integration of hydroxychloroquine with SS-1 as a potential SS treatment was found to significantly increase the Schirmer’s test values for both eyes, and tended to decrease OSDI scores, increase sialoscintigraphy excretion results, and regulate the serum levels of Th17-related pathway cytokines, BAFF, and BCMA. As such, a combination of hydroxychloroquine and SS-1 could usefully complement other therapies aimed at alleviating xerophthalmia and xerostomia in SS sufferers.

### Limitations

This study has several limitations. First, its sample size was not large, and the higher dropout rate was made by crossover analysis methodology with per-protocol analysis, which the results should not be applied as Intention-to-treat analysis. However, the efficacy of Schirmer’s tests of the per-protocol analysis could be examined between the treatment period and placebo period in the same individual SS subject. Second, significant changes in response to SS-1 treatment were not observed for some cytokines; and IL-1β, IFN-α, and IFN-γ could not be detected at all in several patients. These results might be due to the relatively small sample size or the relatively short treatment time. Third, the patients who were receiving biological agents such as rituximab were instructed to discontinue such treatment 1 month prior to their enrollment in our trial, but such agents can have effects that last 3–6 months. That being said, none of the patients who received biological agents before enrolling in the present trial completed it, so in the event, there were no potential confounding effects. Nevertheless, the exclusion criteria in future trials of this kind should include treatment with biological agents. Finally, we did not evaluate the effects of treatment during follow-up, but this was because our crossover trial design enabled us to evaluate the difference between the treatment period and the placebo period in the same individual.

## Conclusion

Based on BioMAP results, we performed a clinical trial to evaluate the therapeutic efficacy of SS-1, and found that it could significantly improve ocular symptoms in both eyes, as indicated by Schirmer’s test results, and also led to significant reduction in daily artificial tear use. SS-1 also regulated patients’ levels of BAFF and BCMA. Importantly, few minor AEs were observed during the trial period. These results suggest that oral SS-1 administration should be considered as an alternative therapy for alleviating xerophthalmia in patients with SS. However, the mechanisms underlying these beneficial effects of SS-1 require further elucidation.

## Data Availability

The original contributions presented in the study are included in the article/Supplementary Material, further inquiries can be directed to the corresponding author.

## Glossary

AECG: American-European Consensus Group
AEs: adverse events
BECs: bronchial epithelial cells
CD: cluster of differentiation
CGMH: Chang Gung Memorial Hospital
CIs: confidence intervals
CMUH: China Medical University Hospital
DFs: dermal fibroblasts
ESSPRI: European League Against Rheumatism Sjögren’s Syndrome Patient Reported Index
OSDI: Ocular Surface Disease Index
PBMCs: peripheral blood mononuclear cells
SAEs: serious adverse events
sIL-17A: secreted interleukin-17A
SS: Sjögren’s syndrome
SSDQ: Sjögren’s syndrome Dry Mouth Symptom Questionnaire
VECs: venular endothelial cells
