# Supplementary material for: Herbal Formula SS-1 Increases Tear Secretion for Sjögren’s Syndrome
Source: Front Pharmacol. 2021 Sep 24;12:645437. doi: 10.3389/fphar.2021.645437 (PMC8498214; doi:10.3389/fphar.2021.645437)
Supplement: Supplementary file 1 [file DataSheet1.docx]

**Supplementary Table S1. The summary of 12 systems from BioMAP® Diversity PLUS panel.**

| **Symbol** | **System** | **Cell Types** | **Description** | **Protein Biomarker Readouts** |
| --- | --- | --- | --- | --- |
| **3C** | Th1 Vasculature | Venular endothelial cells | The Th1 Endothelium (3C) system models vascular inflammation of the Th1 type, an environment that promotes monocyte and T cell adhesion and recruitment and is anti-angiogenic. This system is relevant for chronic inflammatory diseases, vascular inflammation and restenosis. | MCP-1, VCAM-1, TM, TF, ICAM-1, E-selectin, uPAR, IL-8, MIG, HLA-DR, Proliferation, SRB |
| **4H** | Th2 Vasculature | Venular endothelial cells | The Th2 Endothelium (4H) system models vascular inflammation of the Th2 type, an environment that promotes mast cell, basophil, eosinophil, T and B cell recruitment and is pro-angiogenic. This system is relevant for diseases where Th2-type inflammatory conditions play a role such as allergy, asthma, and ulcerative colitis. | MCP-1, Eotaxin-3, VCAM-1, P-selectin, uPAR, SRB, VEGFRII |
| **LPS** | Monocyte Activation | PBMC/Venular endothelial cells | The Monocyte Activation (LPS) system models chronic inflammation of the Th1 type and monocyte activation responses. This system is relevant to inflammatory conditions where monocytes play a key role including atherosclerosis, restenosis, rheumatoid arthritis, and other chronic inflammatory conditions, as well as metabolic diseases. | MCP-1, VCAM-1, TM, TF, CD40, E-selectin, CD69, IL-8, IL1-α, M-CSF, sPGE2, SRB, sTNFα |
| **SAg** | T Cell Activation | PBMC/Venular endothelial cells | The T Cell Activation (SAg) system models chronic inflammation of the Th1 type and T cell effector responses to TCR signaling with costimulation. This system is relevant to inflammatory conditions where T cells play a key role including organ transplantation, rheumatoid arthritis, psoriasis, Crohn's disease and multiple sclerosis. | MCP-1, CD38, CD40, E-selectin, CD69, IL-8, MIG, PBMC Cytotoxicity, Proliferation, SRB |
| **BT** | B and T Cell Autoimmunity | B cells/PBMC | The B and T Cell Autoimmunity (BT) system models T cell dependent B cell activation and class switching as would occur in a germinal center. This system is relevant for diseases and conditions where B cell activation and antibody production are relevant. These include autoimmune disease, oncology, asthma and allergy. | B cell Proliferation, PBMC Cytotoxicity, Secreted IgG, sIL- 17A, sIL-17F, sIL-2, sIL-6, sTNFα |
| **BF4T** | Lung Disease | Bronchial epithelial cells/ Dermal fibroblasts | The Lung Disease (BF4T) system models lung inflammation of the Th2 type, an environment that promotes the recruitment of eosinophils, mast cells and basophils as well as effector memory T cells. This system is relevant for allergy and asthma, pulmonary fibrosis, as well as COPD exacerbations. | MCP-1, Eotaxin-3, VCAM-1, ICAM-1, CD90, IL-8, IL1-α, Keratin 8/18, MMP- 1, MMP-3, MMP-9, PAI-1, SRB, tPA, uPA |
| **BE3C** | Lung Inflammation | Bronchial epithelial cells | The Lung Inflammation (BE3C) system models lung inflammation of the Th1 type, an environment that promotes monocyte and T cell adhesion and recruitment. This system is relevant for sarcoidosis and pulmonary responses to respiratory infections. | ICAM-1, uPAR, IP- 10, I-TAC, IL-8, MIG, EGFR, HLA-DR, IL1-α, Keratin 8/18, MMP-1, MMP-9, PAI-1, SRB, tPA, uPA |
| **CASM3C** | Cardiovascular Disease | Coronary artery smooth muscle cells | The Cardiovascular Disease (CASM3C) system models vascular inflammation of the Th1 type, an environment that promotes monocyte and T cell recruitment. This system is relevant for chronic inflammatory diseases, vascular inflammation and restenosis. | MCP-1, VCAM-1, TM, TF, uPAR, IL-8, MIG, HLA-DR, IL-6, LDLR, M-CSF, PAI- 1, Proliferation, SAA, SRB |
| **HDF3CGF** | Fibrosis and Inflammation | Dermal fibroblasts | The Fibrosis and Inflammation (HDF3CGF) system models wound healing and matrix/tissue remodeling in the context of Th1-type inflammation. This system is relevant for various diseases including fibrosis, rheumatoid arthritis, psoriasis, as well as stromal biology in tumors. | MCP-1, VCAM-1, ICAM-1, Collagen I, Collagen III, IP-10, I-TAC, IL-8, MIG, EGFR, M-CSF, MMP-1, PAI-1, Proliferation_72hr, SRB, TIMP-1, TIMP-2 |
| **KF3CT** | Psoriasis and Dermatitis | Keratinocytes/ Dermal fibroblasts | The Psoriasis and Dermatitis (KF3CT) system models cutaneous inflammation of the Th1 type, an environment that promotes monocyte and T cell adhesion and recruitment. This system is relevant for cutaneous responses to tissue damage caused by mechanical, chemical, or infectious agents, as well as certain states of psoriasis and dermatitis. | MCP-1, ICAM-1, IP- 10, IL-8, MIG, IL-1α, MMP-9, PAI-1, SRB, TIMP-2, uPA |
| **MyoF** | Fibrosis | Lung fibroblasts | The Fibrosis (MyoF) system models the development of pulmonary myofibroblasts, and are relevant to respiratory disease settings as well as other chronic inflammatory settings where fibrosis occurs such as rheumatoid arthritis. | a-SM Actin, bFGF, VCAM-1, Collagen-I, Collagen-III, Collagen-IV, IL-8, Decorin, MMP-1, PAI-1, TIMP-1, SRB |
| **IMphg** | Macrophage Activation | Venular endothelial cells/ Macrophages | The Macrophage Activation (lMphg) system models chronic inflammation of the Th1 type and macrophage activation responses. This system is relevant to inflammatory conditions where monocytes play a key role including atherosclerosis, restenosis, rheumatoid arthritis, and other chronic inflammatory conditions. | MCP-1, MIP-1α, VCAM-1, CD40, E-selectin, CD69, IL-8, IL1-α, M-CSF, sIL-10, SRB, SRB- Mphg |

**Supplementary Table S2. Baseline characteristics of the SS-1 trial for** **patients with primary Sjögren's syndrome**

|  | **Group Placebo－SS-1**  **(N=34)** | **Group SS-1－Placebo**  **(N=31)** | ***p*** |
| --- | --- | --- | --- |
| **Female sex - no. of patients (%)** | 32 (94) | 30 (97) |  |
| **Age, year** | 53.2 ± 11.5 | 51.9 ± 10.8 | 0.775 |
| **Time to diagnosis , year** | 3.7 ± 3.8 | 4.5 ± 3.9 | 0.435 |
| **Conventional medicine** |  |  |  |
| **Hydroxychloroquine** | 231.8 ± 71.6 | 240.9 ± 79.6 | 0.693 |
| **Artificial tear usage (times/day)** | 2.93 ± 2.0 | 3.2 ± 2.3 | 0.665 |
| **Outcome** |  |  |  |
| **Schirmer’s test *(mm)*** |  |  |  |
| **OS (left eye)** | 3.2 ± 4.8 | 2.8 ± 3.6 | 0.728 |
| **OD (right eye)** | 2.8 ± 3.6 | 3.2 ± 5.9 | 0.718 |
| **ESSPRI Score** |  |  |  |
| **Dryness** | 6.4 ± 1.8 | 5.9 ± 2.7 | 0.433 |
| **Fatigue** | 6.2 ± 1.8 | 5.6 ± 2.6 | 0.282 |
| **Pain** | 5.2 ± 2.9 | 3.9 ± 3.1 | 0.084 |
| **OSDI Score** | 50.7 ± 21.7 | 47.2 ± 24.0 | 0.546 |
| **Eyes that are sensitive to light** | 2.5 ± 1.3 | 2.4 ± 1.5 | 0.734 |
| **Eyes that feel gritty** | 2.1 ± 1.4 | 1.9 ± 1.2 | 0.567 |
| **Painful or sore eyes** | 1.4 ± 1.3 | 1.2 ± 1.1 | 0.452 |
| **Blurred vision** | 2.4 ± 1.3 | 1.9 ± 1.3 | 0.135 |
| **Poor vision** | 2.1 ± 1.3 | 1.9 ± 1.4 | 0.713 |
| **Reading** | 2.0 ± 1.3 | 1.9 ± 1.4 | 0.714 |
| **Driving at night** | 1.1 ± 1.4 | 1.1 ± 1.5 | 0.883 |
| **Working with a computer or bank machine (ATM)** | 1.4 ± 1.4 | 1.5 ± 1.5 | 0.836 |
| **Watching TV** | 1.7 ± 1.3 | 1.9 ± 1.3 | 0.538 |
| **Windy conditions** | 2.4 ± 1.3 | 2.1 ± 1.5 | 0.284 |
| **Places or areas with low humidity (very dry)** | 2.3 ± 1.2 | 1.9 ± 1.3 | 0.303 |
| **Areas that are air conditioned** | 2.1 ± 1.3 | 2.0 ± 1.4 | 0.781 |
| **Sialoscintigraphy** |  |  |  |
| **Uptake** | 3.1 ± 1.0 | 3.0 ± 1.1 | 0.918 |
| **Excretion** | 1.9 ± 1.2 | 1.3 ± 1.3 | 0.090 |
| **Laboratory data** |  |  |  |
| **White blood cells *(10^3^ /uL )*** | 5.2 ± 1.2 | 5.0 ± 2.0 | 0.562 |
| **Red blood cells *(10^6^ /uL )*** | 4.3 ± 0.5 | 4.6 ± 0.6 | 0.107 |
| **Hemoglobin *(g/dL )*** | 13.1 ± 1.1 | 13.0 ± 1.2 | 0.713 |
| **Platelet *(10^3^ /uL )*** | 221.3 ± 53.0 | 223.3 ± 50.0 | 0.877 |
| **Aspartate aminotransferase *(U/L)*** | 25.9 ± 7.5 | 24.5 ± 5.0 | 0.383 |
| **Alanine aminotransferase *(U/L)*** | 20.7 ± 8.7 | 19.1 ± 10.1 | 0.493 |
| **Blood urea nitrogen *(mg/dL )*** | 11.8 ± 3.2 | 12.6 ± 4.1 | 0.354 |
| **Serum creatinine *(mg/dL )*** | 0.7 ± 0.2 | 0.6 ± 0.2 | 0.135 |
| **Erythrocyte sedimentation rate *(mm/hr)*** | 18.2 ± 18.7 | 15.3 ± 10.6 | 0.444 |
| **Positive rates — no. of patients (%)** |  |  |  |
| **Rheumatoid factor (RF)** | 9 (26.5) | 9 (29.0) |  |
| **Anti-Ro** | 26 (76.5) | 23 (74.2) |  |
| **Anti-La** | 13 (38.2) | 8 (25.8) |  |
| **Anti-Mitochondrial** | 0 (0) | 2 (6.5) |  |
| **Cytokines *(pg/ml)*** |  |  |  |
| **IL-1β** | 742.27 ± 1141.66 | 627.25 ± 597.40 | 0.847 |
| **IL-17A** | 44.16 ± 114.54 | 198.37 ± 867.05 | 0.346 |
| **IL-18** | 154.99 ± 110.18 | 192.66 ± 259.79 | 0.455 |
| **IL-23** | 59.92 ± 153.37 | 25.97 ± 26.23 | 0.605 |
| **IL-27** | 546.86 ± 948.61 | 992.92 ± 1823.90 | 0.409 |
| **MMP-9** | 35784.92 ± 23563.33 | 44800.64 ± 41695.18 | 0.293 |
| **IFN-α** | 42.13 ± 28.50 | 40.91 ± 37.65 | 0.889 |
| **IFN-γ** | 1296.29 ± 836.36 | 1146.37 ± 647.09 | 0.425 |
| **BAFF** | 304.71 ± 194.00 | 305.64 ± 158.02 | 0.983 |
| **BCMA** | 742.27 ± 1141.66 | 627.25 ± 597.40 | 0.847 |

Abbreviations: SS: Sjögren's syndrome, ESSPRI: EULAR Sjogren's Syndrome Patient Reported Index, OSDI: ocular surface disease index, , MMP-9: matrix metalloproteinase-9, BAFF: B-cell-activating factor, BCMA: B-cell-maturation antigen, IL: interleukin, IFN: interferon
The baseline demographic data and medical conditions were analyzed using ANOVA test for continuous variables, and the baseline characteristics data in the text and tables are expressed as mean ± standard deviation (SD). And there were no significant differences in the baseline characteristics between the two treatment groups.
‖Artificial tear usage: a record which the patient self-reported ranged from 0 to 30 or much more times/per day, the more usage represents more eye dryness.
†Schirmer’s test: an objective evaluation of tear secretion, ranged from 0 to 30 mm, in which less than 5 mm represents dry eye, and more the value represents much more tear secretion).
‡ESSPRI Score: European League Against Rheumatism Sjögren’s Syndrome Patient Reported Index is a patient self-reported questionnaire about dryness, fatigue, and pain, which it ranged from 0 to 10 for each item; while 0 represents no dryness, fatigue or pain; and 10 represents the maximal dryness, fatigue or pain.
§OSDI Score: Ocular surface disease index is a patient self-reported questionnaire containing 12 sub-domains about ocular symptoms, daily activities, and environment factors, which each sub-domain ranged from 0 to 4; while 0 represents none, and 4 represents all the time. The total score of OSDI ranged from 0 to 100, while the higher scores represent greater dry eye disease severity and effect on vision-related functions.
¶ Sialoscintigraphy: an objective evaluation of salivary gland functions, ranged from 0 to 4, bilateral parotid glands were evaluated as 0 represents prominent uptake or excretion; 1 represents moderate uptake or excretion; 2 represents mild uptake or excretion; 3 represents little uptake or excretion; 4 represents no uptake or excretion.

**Supplementary Table S3. The outcome of the SS-1 trial for patients with primary Sjögren's syndrome**

|  | **Placebo group** | **SS-1 group** | ***p*** |
| --- | --- | --- | --- |
| **Schirmer’s test (mm)** | | | |
| **OS (left eye)** | 1.23 (0.18－2.27) | 3.63 (2.58－4.68) | **0.002*** |
| **OS (left eye)^#^** | 3.89 (3.00－4.79) | 5.74 (4.84－6.63) | **0.005*** |
| **OD (right eye)** | 1.41 (0.26－2.55) | 3.72 (2.57－4.87) | **0.006*** |
| **OD (right eye) ^#^** | 4.39 (3.38－5.41) | 6.37 (5.35－7.38) | **0.008*** |
| **ESSPRI Score** | | | |
| **Dryness** | -0.81 (-1.37－-0.24) | -0.95 (-1.51－-0.38) | 0.722 |
| **Fatigue** | -0.94 (-1.57－-0.32) | -0.93 (-1.55－-0.30) | 0.969 |
| **Pain** | -0.73 (-1.52－0.06) | 0.01 (-0.78－0.80) | 0.193 |
| **OSDI Score** | -6.27 (-11.66－-0.89) | -8.84 (-14.23－-3.46) | 0.501 |
| **Eyes that are sensitive to light** | -0.35 (-0.69－-0.01) | -0.32 (-0.66－0.02) | 0.908 |
| **Eyes that feel gritty** | -0.04 (-0.33－0.25) | -0.02 (-0.30－0.27) | 0.920 |
| **Painful or sore eyes** | -0.21 (-0.49－0.08) | -0.07 (-0.36－0.21) | 0.503 |
| **Blurred vision** | -0.26 (-0.58－0.07) | -0.18 (-0.50－0.15) | 0.729 |
| **Poor vision** | -0.32 (-0.71－0.07) | -0.28 (-0.67－0.12) | 0.872 |
| **Reading** | -0.13 (-0.48－0.21) | -0.32 (-0.66－0.02) | 0.445 |
| **Driving at night** | 0.09 (-0.20－0.39) | -0.16 (-0.45－0.14) | 0.227 |
| **Working with a computer or bank machine (ATM)** | -0.22 (-0.54－0.11) | -0.23 (-0.56－0.09) | 0.940 |
| **Watching TV** | -0.14 (-0.41－0.13) | -0.52 (-0.79－-0.25) | **0.048*** |
| **Windy conditions** | -0.23 (-0.53－0.07) | -0.26 (-0.56－0.04) | 0.886 |
| **Places or areas with low humidity (very dry)** | -0.24 (-0.56－0.07) | -0.47 (-0.78－-0.16) | 0.310 |
| **Areas that are air conditioned** | -0.27 (-0.60－0.07) | -0.37 (-0.70－-0.04) | 0.660 |
| **SSDQ Score** | 9.33 (8.41－10.25) | 10.14 (9.23－11.06) | 0.214 |
| **Sialoscintigraphy** | | | |
| **Uptake** | -0.01 (-0.96－0.15) | -0.64 (-1.20－-0.09) | 0.557 |
| **Excretion** | -0.12 (-0.26－0.01) | -0.01 (-0.14－0.12) | 0.227 |
| **Artificial tear usage (times/day)** | -0.36 (-0.96－0.23) | -1.50 (-2.10－-0.90) | **0.010*** |
| **Cytokines *(pg/ml)*** |  |  |  |
| **IL-17A** | 52.03 (-69.28－173.35) | 39.65 (-80.51－159.81) | 0.884 |
| **IL-18** | 34.92 (-7.50－77.35) | 22.20 (-21.28－65.69) | 0.676 |
| **IL-23** | 233.92 (-15.14－482.97) | 55.60 (-383.28－494.49) | 0.398 |
| **IL-27** | -583.69 (-1713.33－545.94) | -285.95 (-1384.35－812.44) | 0.703 |
| **MMP-9** | 13420.00 (-3437.79－30278.00) | -2012.89 (-17033.00－13008.00) | 0.175 |
| **BAFF** | 570.64 (64.41－1076.86) | 523.36 (17.14－1029.59) | 0.895 |
| **BCMA** | 177.97 (127.00－228.95) | 111.36 (60.38－162.33) | 0.069 |

Abbreviations: SS: Sjögren's syndrome, ESSPRI: EULAR Sjogren's Syndrome Patient Reported Index, OSDI: ocular surface disease index, , MMP-9: matrix metalloproteinase-9, BAFF: B-cell-activating factor, BCMA: B-cell-maturation antigen, IL: interleukin, IFN: interferon
The data are presented as mean and 95% confidence interval with crossover analysis, * p<0.05, which these variables were calculated change from baseline.
^#^Schirmer’s test of OS (left eye) and OD (right eye) were analyzed by 2×2 crossover design with original value
‖Artificial tear usage: a record which the patient self-reported, ranged from 0 to 30 or much more times/per day, the more usage represents more eye dryness.
†Schirmer’s test: an objective evaluation of tear secretion, ranged from 0 to 30 mm, in which less than 5 mm represents dry eye, and more the value represents much more tear secretion).
‡ESSPRI Score: European League Against Rheumatism Sjögren’s Syndrome Patient Reported Index is a patient self-reported questionnaire about dryness, fatigue, and pain, which it ranged from 0 to 10 for each item; while 0 represents no dryness, fatigue or pain; and 10 represents the maximal dryness, fatigue or pain.
§OSDI Score: Ocular surface disease index is a patient self-reported questionnaire containing 12 sub-domains about ocular symptoms, daily activities, and environment factors, which each sub-domain ranged from 0 to 4; while 0 represents none, and 4 represents all of the time. And the total score of OSDI ranged from 0 to 100, while the higher scores represent greater dry eye disease severity and effect on vision-related functions.
††SSDQ scores: Sjögren’s Syndrome dry-mouth symptom questionnaire is a patient self-reported questionnaire about dry month using in Taiwan, which it ranged from -10 to 30, in which the scores over 10 represent the efficacy of this drug for improving dry month.
¶ Sialoscintigraphy: an objective evaluation of salivary gland functions, ranged from 0 to 4, bilateral parotid glands were evaluated as 0 represents prominent uptake or excretion; 1 represents moderate uptake or excretion; 2 represents mild uptake or excretion; 3 represents little uptake or excretion; 4 represents no uptake or excretion
